# Sialotranscriptomics of the argasid tick *Ornithodoros moubata* along the trophogonic cycle

**DOI:** 10.1371/journal.pntd.0009105

**Published:** 2021-02-05

**Authors:** Ana Oleaga, Beatriz Soriano, Carlos Llorens, Ricardo Pérez-Sánchez

**Affiliations:** 1 Parasitology Laboratory, Institute of Natural Resources and Agrobiology (IRNASA, CSIC), Cordel de Merinas, Salamanca, Spain; 2 Biotechvana, Scientific Park, University of Valencia, Valencia, Spain; University of Connecticut Health Center, UNITED STATES

## Abstract

The argasid tick *Ornithodoros moubata* is the main vector of human relapsing fever (HRF) and African swine fever (ASF) in Africa. Salivary proteins are part of the host-tick interface and play vital roles in the tick feeding process and the host infection by tick-borne pathogens; they represent interesting targets for immune interventions aimed at tick control.

The present work describes the transcriptome profile of salivary glands of *O*. *moubata* and assesses the gene expression dynamics along the trophogonic cycle using Illumina sequencing.

*De novo* transcriptome assembling resulted in 71,194 transcript clusters and 41,011 annotated transcripts, which represent 57.6% of the annotation success. Most salivary gene expression takes place during the first 7 days after feeding (6,287 upregulated transcripts), while a minority of genes (203 upregulated transcripts) are differentially expressed between 7 and 14 days after feeding. The functional protein groups more abundantly overrepresented after blood feeding were lipocalins, proteases (especially metalloproteases), protease inhibitors including the Kunitz/BPTI-family, proteins with phospholipase A2 activity, acid tail proteins, basic tail proteins, vitellogenins, the 7DB family and proteins involved in tick immunity and defence. The complexity and functional redundancy observed in the sialotranscriptome of *O*. *moubata* are comparable to those of the sialomes of other argasid and ixodid ticks.

This transcriptome provides a valuable reference database for ongoing proteomics studies of the salivary glands and saliva of *O*. *moubata* aimed at confirming and expanding previous data on the *O*. *moubata* sialoproteome.

## Author summary

The soft tick *Ornithodoros moubata* constitutes an important medical and veterinary problem in Africa because, in addition to being the vector of African swine fever, it transmits human relapsing fever (HRF), a hyper-endemic and lethal, but still neglected, tick-borne disease. Effective control of HRF requires eradicating its vector tick from domestic environments. As chemical acaricide application is ineffective against this tick, development of anti-tick vaccines seems the most promising method for tick control. Salivary proteins play essential functions for tick feeding and survival, which convert them in potential antigen targets for the development of tick vaccines. To know which these proteins are, we obtained the salivary transcriptome of *O*. *moubata* females and established, for the first time in a soft tick, the salivary gene transcription dynamics along its trophogonic cycle. Thereby, we have identified numerous genes encoding bioactive proteins essential for tick feeding. This information is essential to drive the selection of candidate antigens for anti-tick vaccine development and evaluate its protective potential in animal immunization trials. These data significantly enlarge the current repertory of known protein-coding sequences from soft tick salivary glands and establish a valuable reference database to improve our knowledge of the *O*. *moubata* salivary proteome.

## Introduction

Ticks are hematophagous ectoparasites that vector a large range of pathogens affecting human and animal health, causing significant production losses worldwide [1,2].

Ticks are classified into two main families, Ixodidae (hard ticks) and Argasidae (soft ticks) with morphological and biological differences between them. Most ixodids are exophilic organisms that stand in soil or vegetation waiting for a suitable vertebrate host. They feed for several days and ingest enormous amounts of blood; after feeding, preimaginal stages moult to the following stage and adult ticks reproduce and die. On the contrary, most argasids are endophilic ticks that live inside the burrows of their wild hosts and colonize human dwellings and domestic animal premises. Argasid tick feeding takes less than 1 hour and, after detachment they moult and reproduce inside their refuges. Adult argasids can feed and reproduce up to 10 times [3].

*Ornithodoros moubata* is an argasid tick widely distributed throughout many countries of central, eastern and southern mainland Africa and Madagascar [4], where it constitutes an important medical and veterinary problem because it transmits lethal diseases such as human relapsing fever (HRF) and African swine fever (ASF) [5,6]. In these countries, *O*. *moubata* is found in nature, associated to warthogs and other wild hosts inhabiting burrows, as well as in anthropic environments, living inside human dwellings and domestic animal premises, which greatly contributes to the transmission and persistence of HRF and ASF in the affected areas. Thus, the presence of this argasid in the anthropic environment makes it difficult to eradicate these diseases from endemic areas. Tick control based on chemical acaricides has raised a number of concerns, including the selection of resistant strains and the accumulation of chemical residues in animal products and the environment. Additionally, their efficacy against *Ornithodoros* ticks is limited because these agents do not reach the ticks inside their refuges [7]. This highlights the necessity of developing alternative methods for tick control, of which vaccination or immunological control is the most promising, environmentally friendly and sustainable ones [8]. A range of antigens induce partial protection against ixodid and argasid ticks [9,10], but it is still necessary to discover new, highly protective antigens.

In the search for new tick protective antigens, scientists are currently targeting the molecules and biological processes specifically evolved by ticks to adapt to their hematophagous lifestyle, namely (i) processes related to host attachment and blood ingestion, including modulation of host defensive responses, which are essentially performed by secreted salivary proteins inoculated to the host in the tick saliva, and (ii) processes related to blood digestion, including nutrient transport and metabolism, haem group and iron management, detoxification and oxidative stress responses, which are carried out by proteins expressed in the midgut [11–16].

Accordingly, an increasing number of recent investigations aimed at the identification of protective antigens from ticks obtained the transcriptome and proteome of the salivary glands and midgut, and the resulting sialomes (transcriptomes and proteomes of the salivary glands) and mialomes (transcriptomes and proteomes of the midgut) have been annotated and inspected for the selection and characterisation of antigenic candidates [17,18].

Most of the studies with salivary glands have been performed in ixodid ticks, and the sialomes of more than 20 ixodid tick species have already been published. In argasids, these studies have been scarcer and, as far as we know, the sialomes of only six species have been published, i.e. *Ornithodoros coriaceus*, *Ornithodoros parkeri*, *Ornithodoros turicata*, *Ornithodoros rostratus*, *Argas monolakensis* and *Antricola delacruzi* [19]. These sialomes have uncovered thousands of protein-coding sequences and large multigene protein families, many of them conserved between both tick families, which underlines that tick sialomes and saliva composition are highly complex and functionally redundant [19]. All these tick salivary protein sequences have been compiled and classified into a recently constructed database (TickSialoFam) [20].

Neither the genome of *O*. *moubata* nor its sialotranscriptome have been sequenced hitherto, and information on its salivary proteins and saliva composition is limited. This information was obtained from a few proteomic studies of its salivary glands/saliva, using different experimental approaches (2D SDS-PAGE, NAPPA technology, LC-MS/MS). In these studies, a significant number of salivary proteins was identified, despite the limitations imposed by the paucity of known tick sequences available at the time when these studies were performed [21–23].

Ixodid and argasid ticks have different feeding strategies. Ixodids take several days to feed and, throughout this time, change the composition of their sialome and saliva several times. This process is known as “sialome switching” and may serve as a mechanism of immune evasion and adaptation to feed on different hosts [20].

Conversely, the rapidly feeding argasid ticks have all the salivary molecules they need to complete feeding already synthetised and stored in the salivary glands before they access the host. After feeding, their protein synthesis machinery must replace all the salivary bioactive proteins consumed during blood ingestion in order to be able to repeat a new trophogonic cycle. The identity of these proteins in *O*. *moubata* and the post-feeding point of time when they are synthetised are currently unknown. We assume that transcripts whose expression increases between two consecutive blood feeding events encode bioactive proteins that are necessary for blood ingestion and hence represent interesting targets for immune interventions aimed at tick control.

Accordingly, the objectives of the current study are to (i) assemble and annotate the sialotranscriptome of *O*. *moubata*, (ii) assess the gene expression dynamics of the salivary proteins along the trophogonic cycle and (iii) characterise the differentially expressed genes at two time points after feeding. Additionally, some particular groups of genes that were abundantly expressed and are functionally related to host attachment, blood ingestion and modulation of host defensive responses have been analysed in more detail because these genes would be encoding bioactive proteins needed for tick blood feeding and consequently they may represent interesting targets for development of vaccines aimed at control of tick infestations and tick-borne disease transmission. This transcriptome provides a valuable database for confirming and expanding previous data of the proteome of *O*. *moubata* saliva (proteome informed by transcriptome).

## Material and methods

### Ethics statement

All protocols involving tick feeding and rabbit manipulation followed the regulations established by the Ethical and Animal Welfare Committee of the IRNASA-CSIC, according to the corresponding EU legislation (Directive 2010/63/EU).

### Tick specimens and salivary glands collection

The *Ornithodoros moubata* ticks were obtained from a pathogen-free laboratory colony, maintained in the IRNASA-CSIC (Salamanca, Spain), which was established in the 1990s from specimens kindly donated by Dr Philip Wilkinson (Institute for Animal Health, Pirbright, UK). Ticks were kept at 28°C, 85% relative humidity, 12 h light/12 h dark photoperiod and regularly fed on New Zealand White rabbits. For tick feeding, rabbits were immobilised with their abdomen facing up and their skin shaved. Ticks were allowed to feed by placing them inside a plastic cylinder fixed to the rabbit skin with surgical tape. Ticks feed during approximately 60 minutes, and after detaching themselves they were collected. The rabbits were not treated with any anaesthetic or tranquilizer drug in order not to interfere with the physiology of the ticks.

Salivary glands (SG) were obtained from newly moulted 3-month-old female ticks in three different physiological states: unfed (SG0) and at 7 and 14 days after feeding (SG7 and SG14, respectively). Tick dissection and salivary gland extraction were performed in cold (4°C) phosphate-buffered saline (PBS) pH 7.4 treated with 0.1% diethyl pyrocarbonate (DEPC), and the SG were immediately stabilised in RNAlater solution (Sigma). For each physiological state, two replicate samples of 20 pairs of SG per sample were collected and used for RNA isolation.

### Total RNA extraction, library construction and sequencing

Library preparation and sequencing were carried out at the Genomics Services of the Fundación Parque Científico de Madrid (Spain) (https://fpcm.es/en/servicios-cientificos/).

The six SG samples (two biological replicates for each physiological state: SG0_1 and SG0_2, SG7_1 and SG7_2, SG14_1 and SG14_2) were processed similarly. Salivary tissue was mechanically disrupted in the TissueLyser II (Qiagen), and total RNA was extracted using the Monarch Total RNA Miniprep Kit" (New England BioLabs) according to the manufacturer´s instruction and including on-column treatment with Turbo DNAse-free (Ambion) to remove any traces of contaminant DNA. Total RNA quality and concentration were assessed in the 2100 Bioanalyzer (Agilent), showing that all samples had RNA integrity number (RIN) values between 7.20 and 8.50.

Next, 1 μg of total RNA from each sample was used as input for library preparation with "NEBNext Ultra II Directional RNA Library Prep Kit for Illumina" (New England BioLabs), following the manufacturer´s recommendations for the Poly(A) mRNA protocol. Fragmentation time was reduced to 10 min in order to recover larger size fragments, which may facilitate the assembly of pair-end reads.

The resulting libraries were validated and quantified in the 2100 Bioanalyzer (Agilent). An equimolecular library pool was made, purified using AMPure XP beads (Beckman Coulter) and titrated by quantitative PCR using the “Kapa-SYBR FAST qPCR kit for LightCycler 480” and a reference standard for quantification. The library pool was denatured and seeded on a NextSeq v. 2.5 flowcell (Illumina), where clusters were formed and sequenced using a "NextSeq 500 High Output kit v. 2.5" (Illumina) in a 2 x 150 pair-end read sequencing run on a NextSeq 500 sequencer (Illumina).

### Pre-processing and transcriptome *de novo* assembly

For all samples, raw reads were converted to FastQ format and subjected to quality control using FastQC (http://www.bioinformatics.babraham.ac.uk/projects/fastqc/). Read quality nucleotides were assessed using as a threshold a PHRED quality score above 30. Reads files longer than 100 nucleotides and with less than 5% sequence indetermination were filtered and trimmed of low-quality data (first 10 nucleotides) using Prinseq [24].

Each sample was assembled *de novo* using Oases [25], applying a k-mer range of 87–97. A merged transcriptome for each physiological state as well as a consensus transcriptome from all six samples were obtained using Minimus2 from the Amos package [26]. Redundant transcripts above a similarity threshold of 95% were eliminated using CD-HIT [27].

### Transcriptome annotation and characterisation

Coding sequences of the consensus transcriptome were searched using the ORFPredictor software [28] and SeqEditor [29], and all ORFs longer than 240 nucleotides (nt) were selected for annotation.

Annotation was performed using the BLASTx and BLASTn programs of the NCBI BLAST package, with an e**-**value< 10^−05^ as cutoff threshold against different databases, such as the NCBI non-redundant sequence database (NR) restricted to Arthropoda [30,31], Swiss-Prot [32] and the genome of *Ixodes scapularis* retrieved from Ensembl [33]. For this, the sequences selected in these databases (21 November 2019) were downloaded and combined in a custom database holding 8,048,569 sequences.

The predicted polypeptides were characterised in the following way. First, functional characterisation including identification of conserved protein domains and protein families according to the Pfam terms [34] included in the Uniprot database and the Interpro database [35], respectively; assignation of Gene Ontology (GO) categories [36] based on Uniprot accessions regarding biological process, molecular function and cellular component categories using the Worksheet tool of the GPRO Suite; metabolic pathways analysis from Kyoto Encyclopedia of Genes and Genomes (KEGG) [37] using the enzyme codes (EC) associated to functional GO categories as queries. Second, topological characterisation, including detection of transmembrane domains, glycosyl-phosphatidyl-inositol (GPI) anchors and signal peptide using, respectively, the following tools: TMHMM [38], PredGPI [39] and signalP-5.0 [40]. Third, antigenicity prediction with Vaxijen 2.0 [41], using a threshold cutoff at 0.5. Topological characterisation and antigenicity prediction were carried out for annotated transcripts only.

### Differential expression and enrichment analyses

To perform differential expression and enrichment analyses between the distinct conditions, raw sequence reads from each library were mapped against the consensus transcriptome, using the mapper Bowtie2 [42]. The average alignment rate was higher than 98% for all libraries, thus confirming the quality of the consensus transcriptome. Corset [43] was applied to hierarchically cluster short transcripts into long genes for downstream analyses, resulting in a cluster file grouping the 80,684 consensus transcripts into 76,194 transcript clusters and a count file summarising the read counts obtained per cluster from each of the six pair-end libraries mapped to the consensus transcriptome. Read counts per transcript were used as input to the EdgeR package [44] to perform three differential expression analyses between physiological states: 7-day fed *ver*sus unfed (SG7 *vs* SG0), 14-day fed *versus* unfed (SG14 *vs* SG0) and 14-day fed *versus* 7-day fed (SG14 *vs* SG7). Transcripts showing a log_2_ fold-change (FC) ≥ or ≤ 1 were considered as differentially expressed when they had an adjusted p-value< 0.05 after False Discovery Rate (FDR) correction applied by EdgeR using the Benjamini-Hochberg method [45].

Gene ontology and metabolic pathway enrichment analyses of the differentially expressed transcripts were performed using the GOseq package [46]. Metabolic pathway enrichment analysis of the differentially expressed transcripts was performed using the Kyoto Encyclopedia of Genes and Genomes (KEGG) database. For this, the enzyme codes (EC) associated to the enriched GO terms were used to download KEGG maps [47] and recover information of the pathways involved, which were annotated using the GPRO suite [48]. Enriched GO terms and metabolic pathways showing adjusted p-values< 0.05 (FDR correction) in the resulting Wallenius distribution were considered significant.

### Pipelines

The protocols and tools for pre-processing, de novo assembly, annotation and differential expression analyses were executed using the DeNovoSeq and the RNAseq tools of the GPRO suite [48].

## Results and discussion

### Sequencing and *de novo* assembly of the salivary transcriptome of *O*. *moubata* females at three distinct physiological conditions

As female ticks is the developmental stage that have to transform the nutrients from the blood meal in new offspring, females were chosen for this study in order to identify and select candidate antigens for the development of vaccines which may induce double deleterious effects, such as an increase in mortality rates and reductions in fecundity and fertility.

In an attempt to assess the gene expression dynamics of the salivary proteins of *O*. *moubata* females along their trophogonic cycle, we prepared six transcriptome samples from salivary glands of females in three physiological conditions (two biological replicates per condition): unfed females (SG0_1, SG0_2) and females at 7 (SG7_1, SG7_2) and 14 (SG14_1, SG14_2) days after blood feeding. Samples were sequenced using illumina RNAseq paired-end technology. The resulting FastQ libraries were processed for quality control and *de novo* assembly, which delivered 70,133 and 84,920 contigs for biological replicates of SG0 (SG0_1, SG0_2), 51,108 and 42,603 for SG7 (SG7_1, SG7_2) and 51,245 and 56,631 for SG14 (SG14_1, SG14_2) (S1 Table). Only reads longer than 100 nucleotides were used in the assembly.

Consensus transcriptomes were built for each physiological state with 60,283, 41,911 and 39,563 contigs for SG0, SG7 and SG14, respectively (Table 1).

**Table 1:** Metrics for de novo transcriptome assembly. (SG0, salivary gland from unfed ticks; SG7 and SG14, salivary gland from ticks at 7 and 14 days after a blood meal respectively).

	SG0	SG7	SG14	Merged
Number of contigs	60,283	41,911	39,563	80,684
Total size of contigs	84,620,350	55,354,425	56,561,536	112,734,597
Longest contig	15,640	17,994	15,564	17,994
Shortest contig	101	101	100	101
% contigs > 1K nt	51.94%	47.03%	52.10%	50.33%
Mean contig size	1,403	1,320	1,429	1,397
N50 contig length	1,965	1,985	2,016	2,041
L50 contig count	13,225	8,442	8,536	16,802
contig %A	26.2	25.95	25.89	26.24
contig %C	23.91	24.14	24.12	23.84
contig %G	23.39	23.57	23.65	23.39
contig %T	26.43	26.27	26.26	26.42
contig %N	0.07	0.07	0.09	0.12
Transcript clusters	-	-	-	76,194
Transcript clusters with ORF ≥240 nt	-	-	-	54,845
Annotated transcripts	-	-	-	41,011
Number of non-redundant accession codes	-	-	-	16,760

To facilitate further comparative analyses, a consensus transcriptome was obtained merging the six assemblies reconstructed de novo. The consensus transcriptome is based on 80,684 transcripts with the following metrics: N50 contig length 2,041 nt, mean contig size 1,397 nt and longest contig size 17,994 nt. Redundancy filtering of these 80,684 transcripts resulted in 76,194 transcript clusters, of which 54,845 had predicted ORFs longer than 240 nucleotides and were selected for functional annotation, characterisation, differential expression and enrichment analyses (Table 1).

### Characterisation and functional annotation: classification according to GO terms, domain families and biological pathways

Blast searching of the 54,845 transcripts against different databases such as the NCBI NR, Swissprot and the *Ixodes scapularis* genome databases allowed the annotation of 41,011 (74.78%) sequences with an e**-**value< 10^−05^; 30,171 of them (73.6%) showed sequence similarity between 60 and 100% (S2 Table). Up to 13,834 sequences (25.2%) did not show significant homology to any sequence in the consulted databases. This set of sequences may represent as yet unknown proteins, but also potentially misassembled sequences of no biological significance or even long non-coding RNAs, which are not easily distinguishable from misassembled ORFs [49,50]. For the 41,011 annotated ORFs, a total of 16,760 non-redundant accession codes were found.

**Fig 1:**
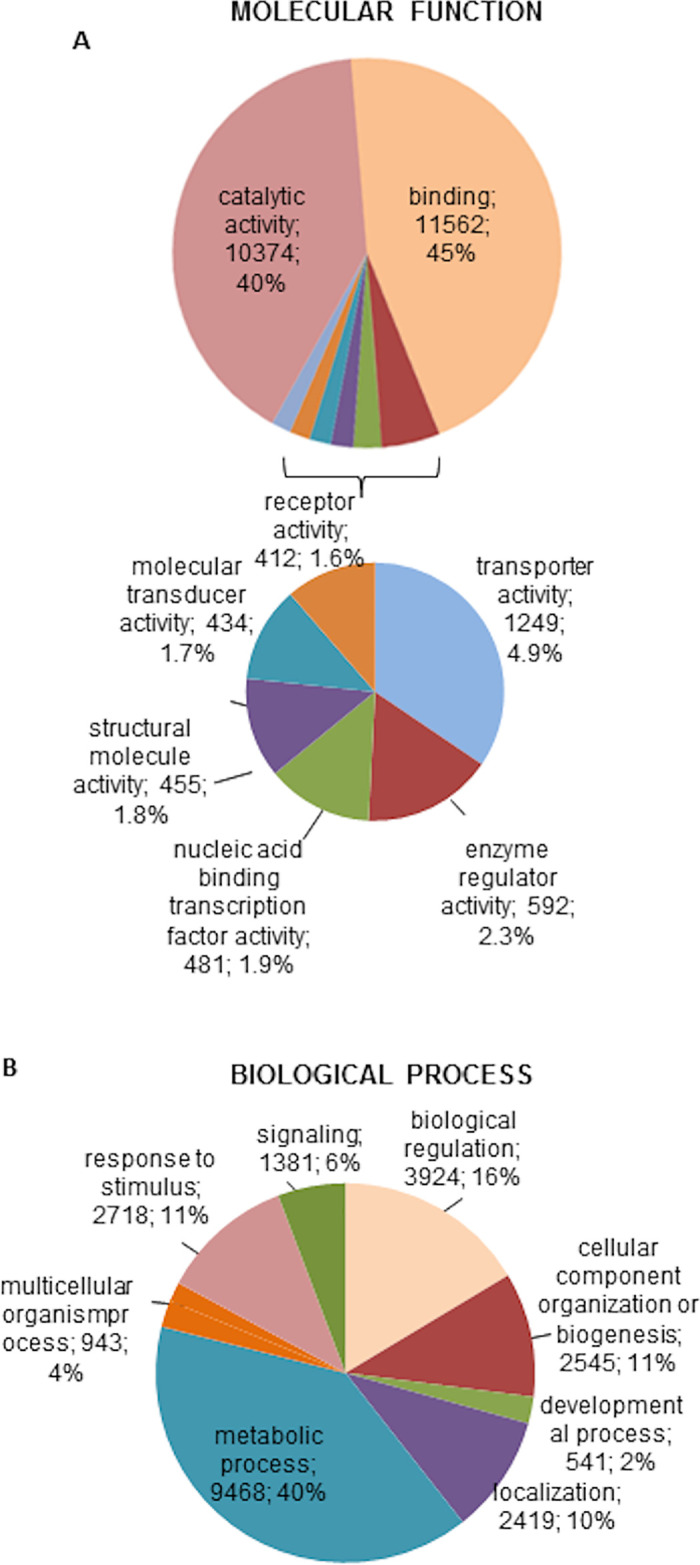
*Ornithodoros moubata* sialotranscriptome. Classification of the annotated transcripts according to the molecular function (A) and biological process (B). Only categories represented by more than 400 genes are included in the pie charts. For each category, the number and percentage of genes are indicated.

The annotated transcripts were functionally characterised using the Gene Ontology (GO), Protein Domain Families (Pfam) and Biological Pathways (KEGG) databases associated to Swissprot annotations. We started by classifying them according to their molecular function and biological process in the GO database. Nearly half of the transcripts (18,096 from 41,011) were assigned GO terms (S2 Table), which included 24,600 biological processes and 25,916 molecular functions. Fig 1A and 1B represents the genes classified according to their molecular function and biological process using level 2 GO terms. The pie charts in Fig 1 include only categories represented by more than 400 genes.

The molecular function categories more abundantly represented were catalytic (n = 10,374) and binding activity (n = 11,562), which together included 85% of the genes. Significantly less represented categories were transporter activity (n = 1,249), enzyme regulator (n = 592), nucleic acid-binding transcription factor (n = 481), structural (n = 455), molecular transducer (n = 434) and receptor activities (n = 412) (Fig 1A). Classification by biological process resulted in eight categories: metabolic processes were the most abundantly represented ones (n = 9,468) followed by biological regulation (n = 3,924), response to stimulus (n = 2,718), cellular component organisation or biogenesis (n = 2,545), localisation (n = 2,419), signalling (n = 1,381), multicellular organism processes (n = 943) and developmental processes (n = 541) (Fig 1B). In general, this classification parallels that reported for some close biological systems such as the sialome of *Haemaphysalis flava* and the mialomes of *O*. *moubata* and *O*. *erraticus* [12,14,51]

Analysis of the transcriptome assigned up to 3,047 unique pfam domains to 21,910 transcripts (S3 Table). The more frequently assigned protein domains were the “RNA recognition motif, (a.k.a. RRM, RBD, or RNP domain)”, “Zinc finger (C2H2 type)”, “Reverse transcriptase (RNA-dependent DNA polymerase)”, “Protein kinase domain”, “WD domain, G-beta repeat” and “Helicase-conserved C-terminal domain” (S3 Table). These domains are frequently and abundantly found in all eukaryotic cells and are involved in a wide range of biological functions including signal transduction, ribosome biogenesis, cell cycle control, intracellular transport, chromatin remodelling, cytoskeletal organisation, apoptosis, development, transcriptional regulation and immune responses [52–55]. These protein *domain* families are also abundantly represented in the mialomes of *O*. *moubata* and *O*. *erraticus* [12,14]. In the hard tick *Haemaphysalis longicornis* a tumour necrosis factor-type zinc finger domain-containing protein was identified that regulates the expression of a defensin molecule, affecting both the humoral and cellular immunity of ticks against bacterial infection [56]. Thus, it is possible that members of this protein family are present in the *O*. *moubata* salivary glands, also playing a role in the innate immunity of these ticks.

To identify active biological pathways in the salivary glands of *O*. *moubata*, the 41,011 annotated sequences were analysed in the KEEG pathway database. Up to 5,977 sequences were included in 103 biological pathways, which were grouped into 13 general classes (S4 Table). The top 30 represented pathways are grouped into 9 classes and include 4,285 enzyme sequences (Table 2). These enzymes are mostly involved in metabolic pathways of lipids (1,252 sequences), nucleotides (863 sequences), carbohydrates (739 sequences), amino acids (669 sequences) and xenobiotics biodegradation (280 sequences). The latter probably play a central role in the detoxification of xenobiotic compounds such as insecticides.

**Table 2:** Top 30 more represented biological pathways in the sialotranscriptome of *O*. *moubata*.

Class	Pathway	Number of Seqs	Seqs in Pathway	Pathway Id
Amino acid metabolism	Arginine and proline metabolism	102	92	map00330
	beta-Alanine metabolism	86	77	map00410
	Glycine, serine and threonine metabolism	88	85	map00260
	Lysine degradation	132	131	map00310
	Tryptophan metabolism	164	164	map00380
	Valine, leucine and isoleucine degradation	97	95	map00280
Carbohydrate metabolism	Amino sugar and nucleotide sugar metabolism	139	139	map00520
	Citrate cycle (TCA cycle)	81	78	map00020
	Galactose metabolism	88	88	map00052
	Glycolysis / Gluconeogenesis	99	99	map00010
	Glyoxylate and dicarboxylate metabolism	98	91	map00630
	Inositol phosphate metabolism	109	95	map00562
	Pyruvate metabolism	125	122	map00620
Lipid metabolism	alpha-Linolenic acid metabolism	175	175	map00592
	Arachidonic acid metabolism	210	210	map00590
	Ether lipid metabolism	187	184	map00565
	Fatty acid degradation	142	100	map00071
	Glycerolipid metabolism	119	113	map00561
	Glycerophospholipid metabolism	250	250	map00564
	Sphingolipid metabolism	169	153	map00600
Metabolism of cofactors and vitamins	Nicotinate and nicotinamide metabolism	71	69	map00760
	Retinol metabolism	102	102	map00830
Metabolism of other amino acids	Glutathione metabolism	87	81	map00480
Nucleotide metabolism	Purine metabolism	572	552	map00230
	Pyrimidine metabolism	291	287	map00240
Signal transduction	Phosphatidylinositol signaling system	135	124	map04070
Translation	Aminoacyl-tRNA biosynthesis	87	85	map00970
Xenobiotics biodegradation and metabolism	Drug metabolism—cytochrome P450	74	74	map00982
	Drug metabolism—other enzymes	95	95	map00983
	Metabolism of xenobiotics by cytochrome P450	111	111	map00980

The purine metabolism pathway included by far the higher number of sequences; this has also been observed for the mialome of *O*. *moubata* [12]. In the context of mialome and blood digestion, it was suggested that nucleotide-metabolizing enzymes such as apyrase, due to its ability to hydrolyse ATP and ADP molecules, would avoid platelet activation and aggregation and help maintaining the fluidity of the blood ingested, in turn facilitating its digestion. The presence of theses enzymes in the saliva would also play an essential role in feeding since they would prevent platelet and leucocyte aggregation and thrombus formation in the feeding site, allowing ticks to complete their blood meal [12,57].

### Differential expression of the sialotranscriptome

After functional characterisation of the sialotranscriptome, we aimed to identify and characterise the *O*. *moubata* salivary genes that were differentially expressed as a function of time after blood feeding.

A number of previous studies in several ixodid species have reported that feeding progression is linked with a temporal transcriptional regulation of salivary gene expression [50,58–60]. For argasids, the information on their sialomes is scarcer, and information related to the dynamics of salivary gene expression and protein synthesis is almost inexistent [19].

Unlike ixodids, which synthesise part of their salivary proteins during feeding, the argasids have all the salivary components that they need for feeding already synthesised, stored and ready for secretion before accessing the host. Accordingly, only basal gene expression can be expected before feeding. After blood ingestion, which in *O*. *moubata* takes around 60 min, the salivary protein synthesis machinery must replace all the bioactive proteins consumed during the blood intake to be ready for the following blood meal.

To know which bioactive proteins are synthesised and when their expression takes place, we investigated salivary gene expression in three time points along the trophogonic cycle: before feeding (SG0, basal condition) and at 7 (SG7) and 14 days (SG14) after blood feeding. Since the trophogonic cycle of females of *O*. *moubata* (feeding-oviposition) at 28°C typically takes 21 days [61], 7 and 14 days post-feeding were selected as intermediate time points.

**Fig 2:**
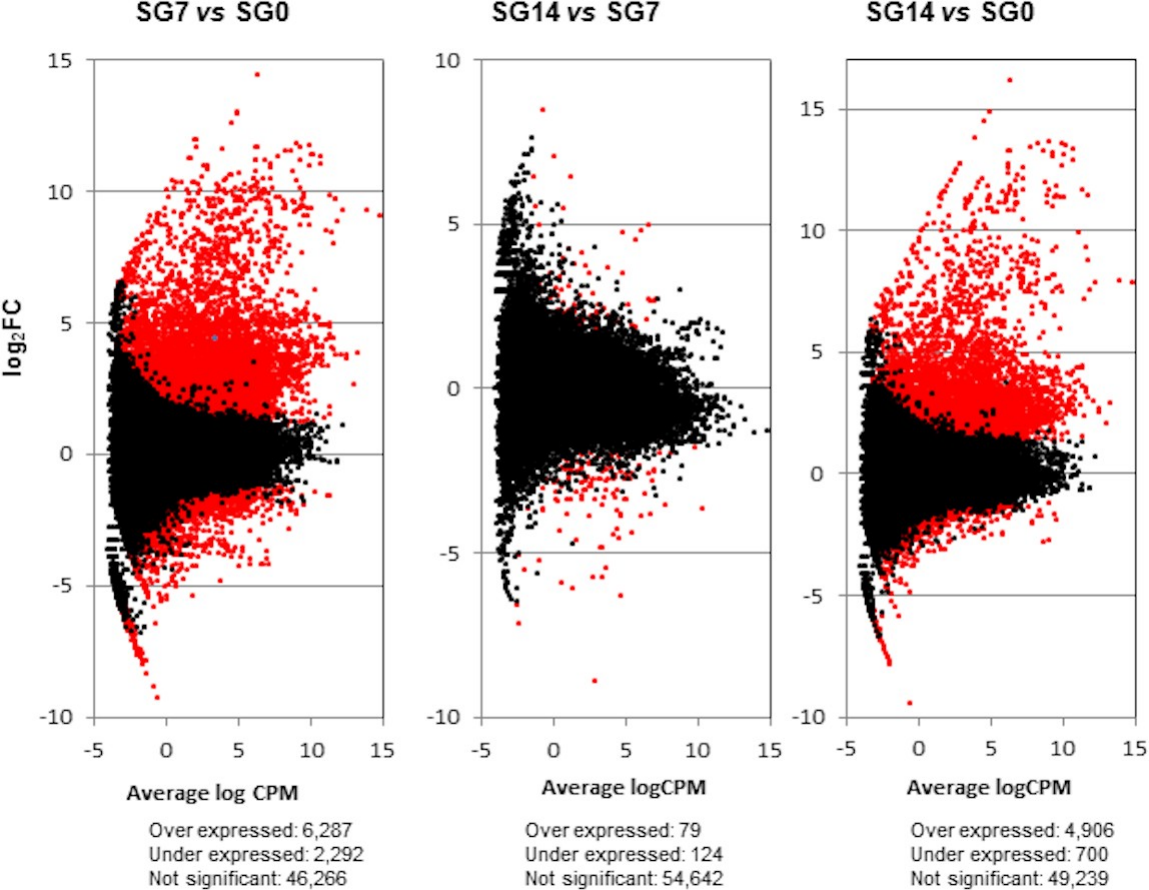
Transcriptome differential expression patterns. Scatterplots (one per each differential expression analysis) representing the log Fold Change (log_2_FC) against average of logCPM per each transcript across each pair of compared samples. Differentially expressed transcripts with FDR < 0.05 and log_2_FC ≥ and ≤ 1 are plotted in red. SG0, salivary glands from unfed ticks; SG7 and SG14, salivary glands from ticks at 7 and 14 days after feeding, respectively. vs, *versus*.

Differential gene expression in the *O*. *moubata* salivary glands was then assessed by comparing the gene expression levels between these different physiological states (SG7 *vs* SG0, SG14 *vs* SG7 and SG14 *vs* SG0) (S5 Table, Fig 2).

**Fig 3:**
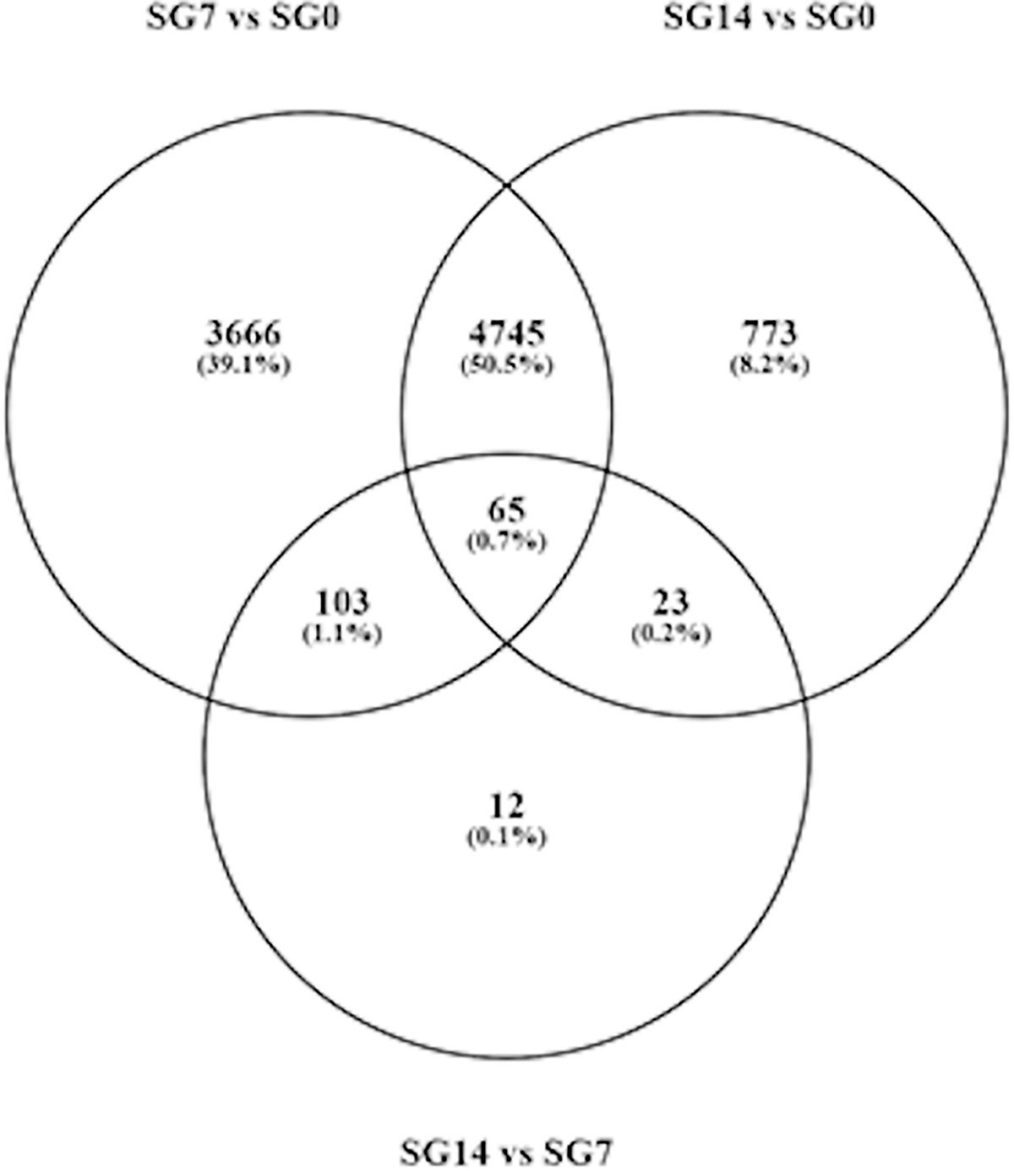
Venn diagram showing the number of differentially expressed genes in each comparison. SG0, salivary glands from unfed ticks; SG7 and SG14 salivary gland from ticks at 7 and 14 days after feeding, respectively. vs, *versus*.

At 7 days after feeding, the gene transcription machinery seems fully active, since at this time point, we observed the highest differential expression in respect to the basal condition (SG7 *vs* SG0): 8,579 differentially expressed transcripts, with 6,287 of them being upregulated (log_2_FC > 1, and FDR < 0.05). Between 7 and 14 days after feeding (SG14 *vs* SG7), there were only slight changes, as only 203 differentially expressed transcripts were detected. Consequently, differential gene expression between basal condition and 14 days after feeding (SG14 *vs* SG0) reflects a situation similar to that observed between basal condition and 7 days after feeding. At 14 days after feeding, up to 5,606 differentially expressed transcripts were detected, of which 4,906 were upregulated (S5 Table, Fig 2). The Venn diagram in Fig 3 represents the number of differentially expressed transcripts in each comparison; 86% of the transcripts that were differentially expressed at 14 days after feeding (SG14 *vs* SG0) had already been differentially expressed at 7 days after feeding (SG7 *vs* SG0).

These results indicate that most expression takes place in the first seven days after feeding and encourage further analyses on salivary gene transcription in shorter time periods, as for example, every 24 hours from detachment time to 7 days post-feeding. This will provide more precise information on gene transcription regulation in each salivary protein family and group, and will show whether the different protein families are differentially expressed over time, as occurs in ixodids [50].

### Enrichment of gene ontologies and metabolic pathways

**Fig 4:**
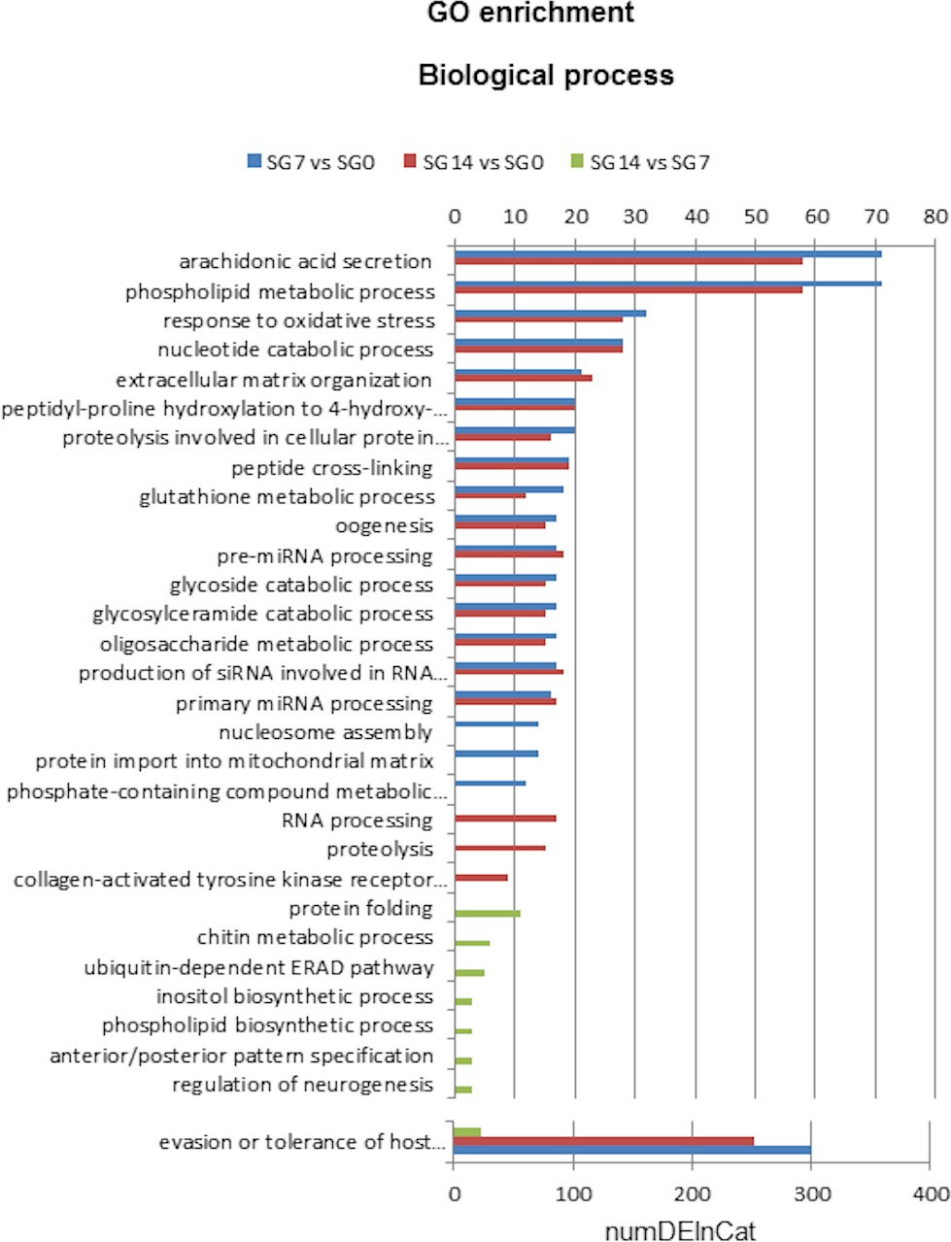
Top 20 GO terms of Biological Process differentially enriched in each comparison: SG7 *vs* SG0, SG14 *vs* SG0 and SG14 *vs* SG7. SG0, salivary glands from unfed ticks; SG7 and SG14, salivary glands from ticks at 7 and 14 days after feeding, respectively. NumDEInCat, number of enriched sequences in each category. *vs*, *versus*.

**Fig 5:**
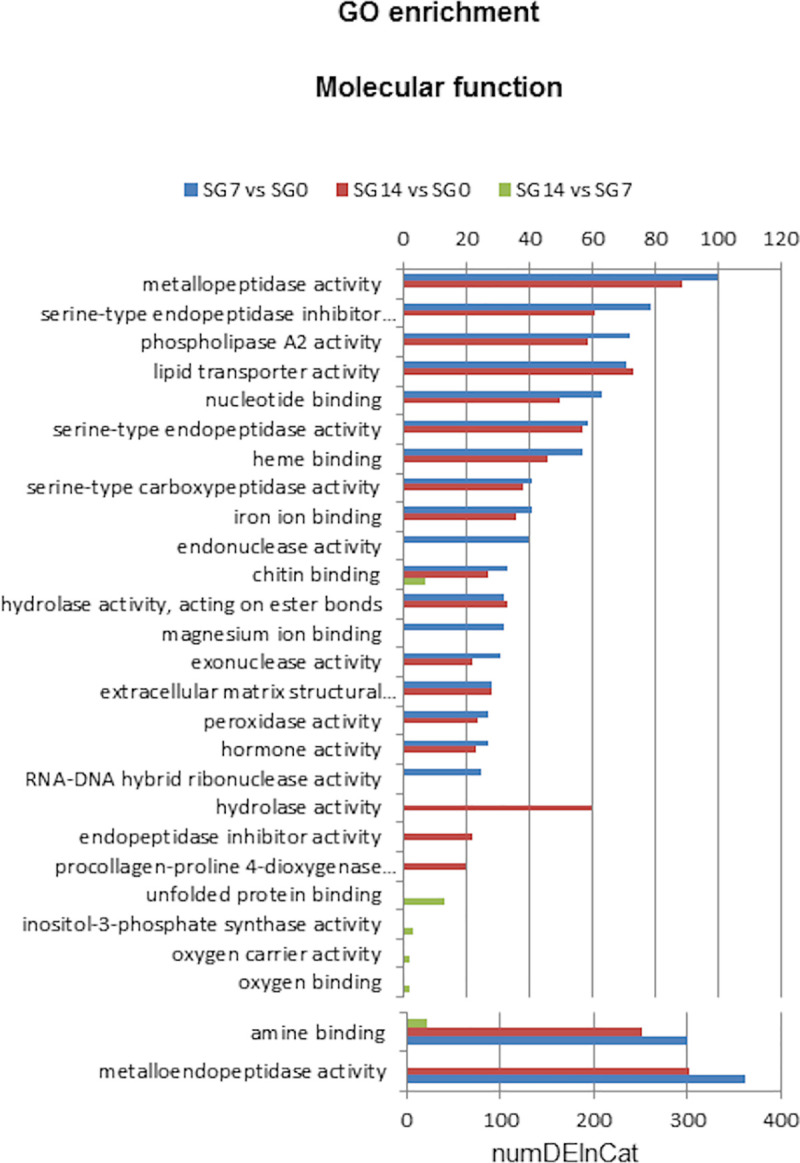
Top 20 Molecular Function GO terms of differentially enriched in each comparison: SG7 *vs* SG0, SG14 *vs* SG0 and SG14 *vs* SG7. SG0, salivary glands from unfed ticks; SG7 and SG14, salivary glands from ticks at 7 and 14 days after feeding, respectively. NumDEInCat, number of enriched sequences in each category. *vs*, *versus*.

Significantly enriched GO terms assigned to the differentially expressed transcripts are compiled in S6 Table. Additionally, Figs 4 and 5 show the significantly overrepresented top 20 Biological Process and Molecular Function GO terms, respectively.

Analyses of GO enrichment revealed differential enrichment of 144 GO terms (FDR< 0.05) in at least one of the three comparisons: 53 biological processes, 78 molecular functions and 13 cellular components (S6 Table).

We found that 39, 29 and 8 biological process GO terms were significantly enriched in comparisons SG7 *vs* SG0, SG14 *vs* SG0 and SG14 *vs* SG7, respectively (S6 Table). Fig 4 shows the overrepresented top 20 biological process GO terms in each comparison. The categories with the highest numbers of upregulated sequences correspond to proteins involved in evasion or tolerance of host defence response, arachidonic acid secretion, phospholipid metabolic processes, response to oxidative stress and nucleotide catabolic processes. These categories reflect several protein families whose genes are abundantly upregulated at 7 and 14 days after feeding, namely the lipocalin family, proteins with phospholipase activity, apyrases and proteins associated with stress responses.

For molecular function GO terms, enrichment analysis revealed that 63, 53 and 6 GO terms were significantly enriched in comparisons SG7 *vs* SG0, SG14 *vs* SG0 and SG14 *vs* SG7, respectively (S6 Table). Fig 5 shows that, at 7 and 14 days after feeding, the molecular functions assigned to a higher number of sequences were metalloendopeptidase activity and amine binding, followed by several peptidase activities, peptidase inhibitor, phospholipase A2 activity and lipid transporter. These functional groups play a prominent role in blood meal intake [62].

The cellular compartment GO terms more enriched in SG7 and SG14 after feeding are sequences assigned to extracellular compartments, most likely related to the synthesis of proteins to be secreted to saliva (S6 Table).

The significantly enriched metabolic pathways of differentially expressed transcripts are shown in S7 Table and in Table 3. This analysis revealed: (i) differential enrichment of 17 biological pathways (FDR< 0.05), grouped in eight classes, in at least one of the three comparisons, and (ii) 13, 10 and 1 biological pathways significantly enriched in comparisons SG7 *vs* SG0, SG14 *vs* SG0 and SG14 *vs* SG7, respectively. This pattern of enrichment along the trophogonic cycle coincides with the patterns observed for GO enrichment and differential gene expression. This is, most pathways are enriched in comparison SG7 *vs* SG0, for which they accrue the highest number of sequences (278); moreover, most of these pathways are also enriched in comparison SG14 *vs* SG0, although accruing a lower number of sequences (181). We also observed that three pathways in the classes “glycan biosynthesis and metabolism” and “xenobiotics degradation” were enriched in comparison SG14 *vs* SG0 only (21 sequences) and that the “inositol phosphate metabolism” was the only enriched pathway in comparison SG14 *vs* SG7 (3 sequences).

**Table 3:** Metabolic pathways differentially enriched in each comparison: SG7 *vs* SG0, SG14 *vs* SG7 and SG14 *vs* SG0. SG0, salivary glands from unfed ticks; SG7 and SG14, salivary glands from ticks at 7 and 14 days after feeding. Num DE seq in Pathway, number of enriched sequences in the pathway. Seqs in Pathway, number of sequences included in the pathway.

Class	Entry	Pathway	Num DE seq in Pathway			Seqs in Pathway
			SG7 vs SG0	SG14 vs SG7	SG14 vs SG0	
Amino acid metabolism	map00330	Arginine and proline metabolism	40	-	32	83
	map00300	Lysine biosynthesis	7	-	-	12
	map00940	Phenylpropanoid biosynthesis	21	-	18	28
	map00350	Tyrosine metabolism	10	-	12	22
Carbohydrate metabolism	map00520	Amino sugar and nucleotide sugar metabolism	22	-	-	82
	map00562	Inositol phosphate metabolism	-	3	-	8
	map00040	Pentose and glucuronate interconversions	4	-	-	6
Energy metabolism	map00910	Nitrogen metabolism	6	-	-	11
	map00190	Oxidative phosphorylation	20	-	-	43
Glycan biosynthesis and metabolism	map00603	Glycosphingolipid biosynthesis—globo series	-	-	4	7
	map00512	Mucin type O-Glycan biosynthesis	-	-	5	10
Lipid metabolism	map00565	Ether lipid metabolism	74	-	59	180
	map00061	Fatty acid biosynthesis	22	-	23	34
	map00140	Steroid hormone biosynthesis	14	-	8	22
Metabolism of cofactors and vitamins	map00670	One carbon pool by folate	6	-	-	9
Metabolism of other amino acids	map00480	Glutathione metabolism	32		17	81
Xenobiotics biodegradation and metabolism	map00980	Metabolism of xenobiotics by cytochrome P450	-	-	12	41

As a whole, the enriched pathways involve the metabolism of amino acids, carbohydrates, lipids, energy, glycan, cofactors and vitamins and xenobiotics, with lipid and amino acid metabolism being the pathways with the highest number of overexpressed sequences (Table 3).

### Main protein groups overexpressed after feeding

We analyse in this section particular groups/families of proteins related to host attachment and blood ingestion, including those involved in the modulation of host defensive responses. In this context, we assume (i) that salivary genes overexpressed after feeding are mainly those encoding the bioactive proteins necessary for blood ingestion and (ii) that these proteins may represent interesting targets for immune or drug interventions aimed at the prevention and control of tick infestations and tick-borne pathogen transmission.

The functional protein groups more abundantly overrepresented in the sialotranscriptome at 7 and 14 days after blood feeding were lipocalins, proteases (especially metalloproteases), protease inhibitors including the Kunitz/BPTI-family, proteins with phospholipase A2 (PLA2) activity, acid tail proteins, basic tail proteins, vitellogenins, the 7DB family and proteins involved in tick immunity and defence. These protein groups and families have also been abundantly found in the sialomes of other ixodid and argasid tick species [20, 63].

**Fig 6:**
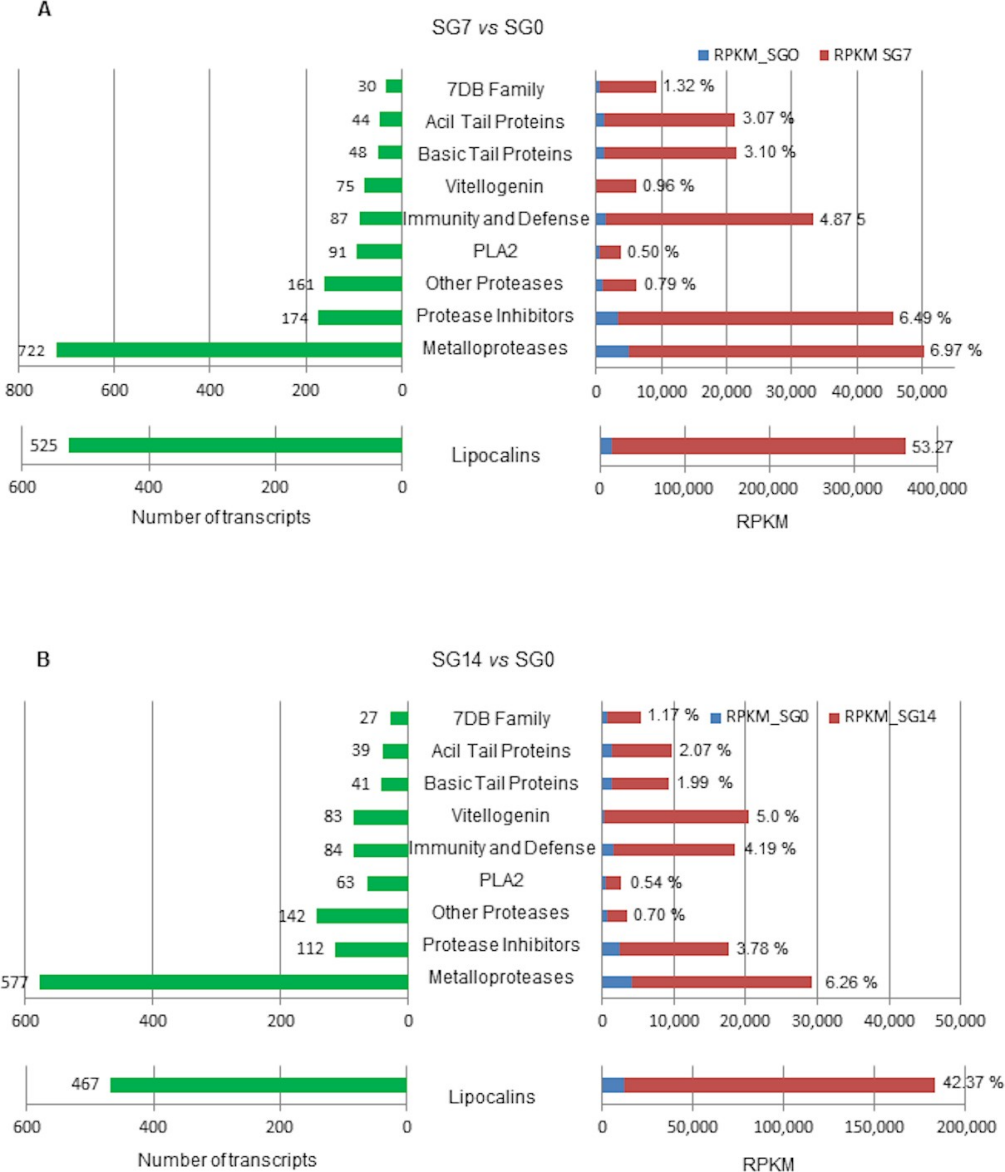
Number of upregulated annotated transcripts in each functional group (green bars) and expression levels in RPKM in comparisons SG7 *vs* SGO and SG14 *vs* SG0 (red bars). Percentages at the red bar ends represent the ratio between the expression level of each group/family and the total expression in RPKM of the whole annotated upregulated transcriptome at 7 days (A) and at 14 days (B). SG0, salivary glands from unfed ticks; SG7 and SG14, salivary glands from ticks at 7 and 14 days after feeding, respectively. *vs*, *versus*.

Fig 6 represents the number of annotated transcripts upregulated in each protein family or group and the corresponding expression level in RPKM at 7 (Fig 6A) and 14 (Fig 6B) days after feeding. Percentages at the bar tips in the right panels of this figure represent the ratio between the expression level of each protein group/family and the total expression in RPKM of the whole annotated upregulated transcriptome at 7 (Fig 6A) or 14 (Fig 6B) days after feeding.

At 7 days after feeding, metalloproteases and lipocalins are the groups that reach both the highest numbers of upregulated transcripts (722 and 525, respectively) and the highest expression levels. Lipocalins are remarkable because of their high expression level, up to 348,330.48 RPKM, which is more than 8 fold the level of the remaining groups and represents 53.3% of expression in the annotated upregulated transcriptome. This result correlates with previous data on the *O*. *moubata* saliva proteome, where lipocalins represented more than 90% of the protein mass [21,23] and compares to the data reported by Mans et al. [64] for the salivary glands of other soft tick species, which showed a good correlation between transcript level and protein abundance. In the remaining protein groups, the highest number of upregulated transcripts was observed for protease inhibitors (178 transcripts), proteases other than metallo (161 transcripts), PLA2 activity (91 transcripts), proteins involved in immunity and defence (87 transcripts) and vitellogenin (75 transcripts). Among these groups, the protease inhibitors and the immunity- and defence-related proteins showed the highest expression levels (Fig 6A).

At 14 days after feeding, the expression pattern of these groups was similar to that of 7 days post-feeding, although with lower transcript numbers and expression levels in each protein group except for vitellogenins, which showed the opposite situation passing from 75 transcripts and 0.96% expression in SG7 to 83 transcripts and 5% expression in SG14.

Thus, what follows is a more detailed analysis of these protein groups/families in the upregulated transcriptome at 7 days after feeding.

#### Lipocalins

Lipocalins are extracellular proteins that share several common recognition properties such as ligand binding, receptor binding and the formation of complexes with other macromolecules [65].

In the saliva of soft ticks, the lipocalin family is one of the more important protein families in terms of member number, high protein expression levels and numerous functions, mainly related to the regulation of host haemostasis and inflammation [21,23,63,66]. These functions are performed by scavenging different agonists including thromboxane A2, leukotriene B4, cysteinyl leukotrienes, histamine, serotonin and the complement component C5, which results in the inhibition of host haemostatic and defensive responses including platelet and neutrophil aggregation, vasoconstriction, complement activation and histamine-mediated inflammation [23,67–69].

In the current sialotranscriptome, up to 297 transcripts have been annotated as lipocalins, salivary lipocalins or salivary secreted lipocalins, 103 transcripts as moubatins, 114 transcripts as TSGP4 and 11 transcripts as golgi-destined proteins, which demonstrates the importance of these proteins for *O*. *moubata* (Table 4).

**Table 4:** Lipocalins: transcripts annotated and differentially upregulated in the *O*. *moubata* sialotranscriptome 7 days after feeding (SG7 *vs* SG0). The number of transcripts, the average expression level (RPKM) in each physiological condition (SG0, SG7) and the logFC value range reached by the upregulated transcripts are shown.

Description	Accession	N° transcripts	RPKM SG0	RPKM SG7	logFC
Golgi-destined protein	ABR23375	11	208.00	1,348.73	1.63–3.81
Lipocalin	ABI52661, ABI52807	17	205.32	1,488.39	2.59–8.60
Moubatin	AAA29432, ABR23347, Q04669	103	6,083.70	81,478.19	1.57–6.21
Salivary lipocalin	ABR23357, ACB70348 ACB70380, ACB70384 ADK94457, ABR23394	275	2,961.30	220,424.70	1.24–10.13
Salivary secreted lipocalin	ACB70358	5	579.19	5,278.44	1.83–3.28
TSGP4	AAN76831, AGJ90348	114	2,931.96	38,312.02	1.33–7.12

Moubatins form a clade that comprises several lipocalins playing diverse functions. Well known members of this clade include moubatin and the complement pathway inhibitor (OmCI) from *O*. *moubata*. Moubatin inhibits platelet aggregation by scavenging of thromboxane A2 (TXA2), whereas OmCI binds to the C5 complement component blocking complement activation; both of them inhibit neutrophil aggregation by scavenging leukotriene B4 (LTB4) [68,70,71]. Lipocalin TSGP4 was first described in *Ornithodoros kalaharensis* and belongs to a clade of cysteinyl leukotriene scavengers which prevent oedematous inflammatory reactions at the tick bite site [67]. More recently, an orthologue of TSGP4 was found in *O*. *moubata* saliva, and its cDNA coding sequence was cloned and characterised [23,69]. The golgi-destined protein has also been found in the sialome of *Ornithodoros parkeri* [66]. The function of this protein in tick saliva is currently unknown, but it is included to the lipocalin family of the InterPro database (IPR002970) and is annotated in the UniProt database as an amine-binding protein. In this context, it is tempting to relate it to the clade of biogenic amine scavenger lipocalins, which function as inhibitors of inflammation by scavenging histamine and serotonin from the tick feeding site [64,72].

#### Salivary proteases

Numerous transcripts were annotated as enzymes with protease activity in the *O*. *moubata* sialotranscriptome. Close to 82% of them are metalloproteases (722 transcripts) and the remaining ones are serine proteases, cysteine proteases, or proteases with an unknown mechanism (161 transcripts) (Table 5, Fig 6A).

**Table 5:** Metalloproteases: transcripts annotated and differentially upregulated in the *O*. *moubata* sialotranscriptome 7 days after feeding (SG7 *vs* SG0). The number of transcripts, the average expression level (RPKM) in each physiological condition (SG0, SG7) and the logFC value range reached by the upregulated transcripts are shown.

Description	Accession	N° transcripts	RPKM SG0	RPKM SG7	logFC
A disintegrin and metalloproteinase with thrombospondin motifs	KFM57926	1	0.62	2.65	2.09
Angiotensin-converting enzyme	B7PXL6, B7PNY9, OQR78107 OQR78107, XP_013773749 XP_023220404, EEC08311	21	43.87	400.68	1.56–3.12
Carboxypeptidase	B7P3H3, XP_028163405 XP_013793241	3	15.24	38.07	1.29–2.77
CNDP dipeptidase	B7PIM5	7	9.09	26.99	1.33–1.90
Endothelin converting enzyme	B7PK74, XP_020279873 RZC36911, B7Q9P6, KFM82655 XP_022902734, XP_022118453 XP_026486430, XP_018573336 XP_013193396, B7PLU3, AMO02506 XP_017752722, XP_003707770	16	105.71	619.69	1.15–5.07
GL13072	EDW26543	1	15.70	84.42	2.43
Hypothetical secreted protein	ACB70342	212	688.88	3,767.41	1.25–6.16
Membrane metallo-endopeptidase-like 1	KFM82803, RWS06231, RZC42385	4	4.76	40.26	2.01–4.81
Metalloprotease	BAE00066, BAF43574, BAE72661 BAF43575, BAE72663, BAF44944 B7PPE9, B7Q2B8, ABR23495 B7Q2C1, B7Q4I3	44	425.11	6,842.95	1.15–5.42
Metalloprotease 2	AIE44748, AIE44751	21	163.92	1,108.60	2.32–3.62
Metalloprotease 3	AIE44749	15	197.94	1,567.43	2.57–3.55
Metalloprotease 4	AIE44755	3	11.48	122.85	2.31–4.08
Metalloproteinase	ABD66751, ABI52652, ABI52815 ABI52662, ABI52680, ABI52714 ABI52712, ABI52719, ABI52738 ABI52747, ABI52789	102	1,131.51	11,352.02	1.37–2.94
Metis1 protein	CAO00625	10	74.22	913.90	1.67–4.43
Metis2 protein	CAO00626	15	50.18	225.53	1.56–2.79
Metis3 protein	CAO00627	16	82.34	346.09	1.43–3.00
Metis4 protein	CAO00628	20	295.52	2,477.63	1.76–3.99
Metis5 protein	CAO00629	5	18.01	115.70	1.92–3.26
Neprilysin	DAA34245, OTF73139, B7PL32 KZS21047, B7Q3V5, B7Q6H6 B7QAF9, EEC07304, XP_018565161 XP_023229763, XP_022255642 XP_023211936, XP_024080796 XP_023321543, RWS05424, ARK20047 W4VS99, XP_019868485 XP_019874967	58	549.06	3,012.68	1.59–9.81
Peptidase	KRT86698	1	0.67	3.12	2.21
Uncharacterized proteins	B7QEM8, B7QM92, B7PKP5, B7Q5E1 B7PA58	27	318.12	4,586.34	1.40–4.91
Salivary gland metalloprotease	DAA34197, DAA34198, B7P187 DAA34210, DAA34252, DAA34264, ISCW014977, ISCW023633, EEC18166, EEC19961, AAZ39659, AAZ39660	38	200.55	1,195.96	1.26–4.74
Secreted metalloprotease	B7QM91, AAM93652 AAM93653, AAT92201	16	208.36	4,530.69	2.61–4.95
Venom metalloproteinase	XP_018494555, ARK20037	3	2.46	9.84	1.50–8.04
Zinc metalloprotease	XP_002433213, ACB70344	63	284.22	2,109.17	1.25–6.16

Metalloproteases constitute a family of proteins that require a metal ion for catalysis, and they are usually found in tick saliva and other tissues. Metalloproteases are essential for different tick biological functions such as inhibiting blood clotting by degrading fibrinogen and fibrin, degrading extracellular matrix proteins, inhibiting host tissue repair via anti-angiogenic activity and facilitating blood feeding [73,74].

Metalloproteases are one of the enzyme classes most abundantly represented in the saliva of *O*. *moubata*, as occurs in other argasid species [63]. Up to 722 transcripts were annotated as different metalloproteases, which represent 6.97% of expression in the upregulated sialotranscriptome (SG7 *vs* SG0) (Fig 6A).

Table 5 summarises the metalloproteases annotated, their expression levels in reads per kilobase per million (RPKM) of each gene [75] and the log_2_FC at 7 days after feeding. They represent a wide repertory of enzymes, most of which play functions related to vascular biology and maintenance of haemostasis, such as, for example, a disintegrin and metalloproteinase with thrombospondin motifs (ADAMTS), angiotensin-converting enzyme, endothelin-converting enzymes, several metis proteins and neprilysins (Table 5).

ADAMTS belongs to a family of proteins that contain both a disintegrin and a metalloprotease domain, which play diverse roles in tissue morphogenesis, inflammation and vascular biology. The ADAMTS1 interacts with the vascular endothelium and produces vasodilatation and an enhancement of vascular permeability, which may increase blood flow to the feeding lesion and facilitate tick feeding [76]. The angiotensin-converting enzyme, a member of the M2 metalloprotease family, plays a functional role in the regulation of blood pressure; as it can degrade bradykinin, it may also avoid pain and itching during tick feeding [77]. Endothelin-converting enzymes hydrolyse large endothelins into the smaller vasoactive endothelins, contributing to blood pressure regulation [78]. Metis metalloproteases are a protease family identified in the hard tick *Ixodes ricinus* and suggested to play several functions related to fibrinolysis, inhibition of wound healing and blood meal success [79,80]. Neprilysins is a family involved in proteolytic activity to several peptides and proteins including gelatine, fibrinogen and fibronectin, which could help regulate host inflammatory and immune responses [81]. They are poorly characterised in invertebrates, but are being increasingly detected in the sialomes of ticks [74,82].

We also consider as a putative metalloprotease the 212 transcripts that were annotated as “hypothetical secreted protein” of *Ornithodoros coriaceus* (ACB70342) (Table 5). This protein is still uncharacterised, but it showed sequence identity > 50% with a putative tick metalloprotease of *Ornithodoros turicata* (A0A2R5L6M4) in Blast searching of the Uniprot database.

Among the non-metallo proteases up to 30 upregulated transcripts were annotated as cysteine proteases, 113 transcripts as serine proteases and 19 transcripts as proteases without known mechanism (Table 6).

**Table 6:** Proteases: transcripts annotated and differentially upregulated in the *O*. *moubata* sialotranscriptome 7 days after feeding (SG7 *vs* SG0). The number of transcripts, the average expression level (RPKM) in each physiological condition (SG0, SG7) and the logFC value range reached by the upregulated transcripts are shown.

Description	Accession	N° transcripts	RPKM SG0	RPKM SG7	logFC
***Cysteine proteases***					
Calcium-dependent cysteine protease	EEC07120, DAA34529	2	1.24	11.60	3.18–3.58
Calpains	XP_028967496, XP_023248422 OQR72424, XP_026481915 XP_013773834, XP_022710373 XP_023215299	7	18.12	160.66	1.30–4.24
Cathepsin B	ACF35525	2	9.05	30.96	1.66–1.84
Cathepsin L	ALQ43545, PRD19376 XP_011297801, QBB68764 QBB68764	6	111.74	373.79	1.47–4.46
Hypothetical protein C7M84_018698	ROT63422	1	3.05	21.35	2.8
Secernin 1, 2 and 3	KFM62462, XP_022251095 XP_022670118	12	13.22	148.74	1.42–4.26
***Serine proteases***					
Carboxypeptidase	B7QF76	17	10.13	77.52	1.20–2.94
Factor D-like protein	AAN78224	3	28.53	104.93	1.71–1.99
Furin-like protease 1	XP_015927741, XP_021002681 XP_021002681, RWS09190 XP_015790166	5	9.07	23.40	1.24–1.46
Ixominogen	AML23866	6	1.13	18.57	2.91–4.52
Limulus clotting factor C	B7PD52, AII02148	16	18.05	73.73	1.74–2.76
Lysosomal protective protein	XP_013784602 XP_023233630	9	5.05	75.28	3.38–4.25
Mannan-binding lectin serine protease 1-like	OQR79970	1	1.21	20.21	4.05
Microsomal signal peptidase	ABI52785	2	47.96	136.38	1.44–1.52
Retinoid-inducible serine carboxypeptidase	ODN00453, KFM77940 XP_013787581	16	30.01	107.89	1.20–2.25
Scpep1	PRD34546	2	4.06	14.30	1.62–1.95
Secreted salivary gland peptide	B7PC21, B7PPZ7	3	23.33	83.07	1.66–1.99
Serine carboxypeptidases	DAA34155, B7PJ32, B7QF79, B7PJ51, XP_022201600	14	159.29	1,183.95	1.92–2.64
Serine proteases	QCT84046, QCT84046, B7QLM5 AAQ82934, BAH03485	8	12.97	77.36	1.23–6.78
Testisin-like	XP_022649100	1	0.09	1.21	3.69
Transmembrane protease serine 9	KFM57227	9	22.22	92.56	1.97–2.27
Trypsin-1	XP_003397616	1	6.77	28.18	2.06
***Others***					
Aminopeptidase N	XP_023224464	1	0.07	0.42	2.56
ATP-dependent Clp protease	ISCW021455-PA	1	13.49	32.04	1.23
M20 domain-containing peptidase	B7P153, B7PPI0 KFM60942 XP_023224767	13	21.42	80.53	1.30–2.23
Protease-associated domain-containing protein of 21 kda	B7PE94	1	4.41	10.07	1.19
Signal peptidase complex	XP_013788579, XP_013788579 B7PXW8	3	132.39	618.26	1.74–2.34

Cysteine proteases include several calcium-dependent cysteine proteases such as cathepsins B and L and calpains. Cathepsins B and L were found upregulated after feeding and involved in blood digestion in the midgut of argasid ticks [12,14,83]. There is no information on the functions of cathepsins B and L in saliva during blood ingestion, but these enzymes are upregulated in the sialome of ixodid ticks during feeding, particularly in male ticks, suggesting functions related to tick mating and reproduction [50,84].

Serine proteases are usually identified in argasid and ixodid sialomes, where they facilitate blood feeding by blocking several host processes such as blood coagulation, inflammation and fibrinolysis [23,50,74].

#### Protease inhibitors

The saliva of blood-feeding parasites is a rich source of protease inhibitors that help overcoming the host defences during host-parasite interactions [85].

Protease inhibitors are abundantly represented in the saliva of hematophagous arthropods, which reflects their importance in facilitating blood intake as antihaemostatic and antiinflammatory factors [63,74]. The Kunitz-BPTI family of serine protease inhibitors is the best known. They possess one or several domains and specifically target thrombin and activated coagulation factor X (FXa), although they may interfere with other haemostatic functions [86].

A total of 174 transcripts were annotated as protease inhibitors in the upregulated *O*. *moubata* sialotranscriptome, among which those containing Kunitz domains were the most numerous (Table 7). Some of these Kunitz domain-containing proteins have already been functionally characterised in *O*. *moubata* and in other argasid tick species. Namely, two anticoagulants, ornithodorin, a two-Kunitz-domain thrombin inhibitor from *O*. *moubata* [87], and the tick anticoagulant peptide (TAP) from *O*. *kalaharensis* (one domain), which inhibits the blood coagulation factor FXa [88], and three disintegrin-type inhibitors of platelet aggregation, known as disaggregin (*O*. *moubata*), savignygrin (*O*. *kalaharensis*) and its orthologue in *O*. *moubata*, mougrin [9,86,89] (Table 7).

**Table 7:** Protease inhibitors: transcripts annotated and differentially upregulated in the *O*. *moubata* sialotranscriptome 7 days after feeding (SG7 *vs* SG0). The number of transcripts, the average expression level (RPKM) in each physiological condition (SG0, SG7) and the logFC value range reached by the upregulated transcripts are shown.

Description	Accession	N° transcripts	RPKM SG0	RPKM SG7	logFC
***Kunitz domain-containing proteins***					
BPTI/Kunitz domain-containing protein	PRD26467, XP_022256442	2	5.74	110.04	3.34–5.90
Disagregin	P36235	9	288.30	7,876.44	2.15–5.05
Dual kunitz salivary protein	ABR23474, ACB70326 ACB70328, ACB70330	23	963.30	10,876.82	1.71–5.17
Hypothetical proteins	CAB55816, RWS20793 OTF84081	4	4.54	66.03	1.63–4.60
Kunitz domain-containing salivary protein	ABI52641, ABR23431 XP_022256441, XP_015432026 XP_023714180	27	370.03	4,089.13	1.54–5.82
Microlepidin-1 precursor	ADV40356	3	173.48	810.45	2.13–2.24
Mougrin	AGJ90345	3	110.22	1,095.61	1.57–3.36
Ornithodorin	P56409	4	104.49	991.02	169–3.27
Papilin	XP_014217269, XP_026818118 XP_026818119, XP_027848886 ADK62391, KFM65460 XP_022257666, XP_023226201 XP_022247907, XP_023211824 XP_023211883, XP_026316271	24	291.48	4,149.39	1.17–4.36
Protease inhibitor carrapatin	P81162	2	13.35	57.44	2.06–2.17
Putative secreted protease inhibitor	AAM93648, AAY66723	2	15.91	347.27	2.45–4.59
Savignygrin	AAM54047	5	67.38	503.90	2.06–3.19
Tick anticoagulant peptide (TAP)	P17726	10	220.12	6,407.68	2.13–5.24
Tissue factor pathway inhibitor	XP_013773410, XP_013773410 XP_015929125	4	56.59	939.47	2.83–4.24
Venom kunitz type-like peptide Vf1	ARB50089	7	25.74	464.44	2.05–4.47
***Others***					
Alpha-2-macroglobulin precursor	AAN10129	11	91.85	299.02	1.49–1.92
Carboxypeptidase inhibitor	PRD20074, Q5EPH2 XP_013781772, XP_022249736	5	7.05	375.75	1.68–10.11
Chymotrypsin inhibitor	KOC63716, ACF57858 XP_011166052, XP_015435639 XP_024226275, XP_029160407 P83516	10	7.60	701.68	1.26–4.67
Cystatin-2	AAS55948	4	102.64	257.65	1.27–1.36
Kappapi-actitoxin-Avd3b-like	XP_022817328	2	0.30	5.92	3.43–4.93
Kazal-type serine protease inhibitor domain protein	DAA34653	1	76.85	662.44	3.11
Pregnancy zone protein-like	XP_022257127	2	22.76	72.25	1.63–1.68
Putative thyropin precursor	AAS01022.1	5	211.45	864.79	1.48–2.12
Serine protease inhibitor	AHC98666, ADV40370 B7PH24, B7PG23	5	54.64	376.92	1.25–3.91

Up to 24 transcripts were annotated as different papilins. These are multi-Kunitz-domain proteins of the extracellular matrix that regulate the formation of basement membranes [90]. Papilins have recently been identified in the sialome of *O*. *rostratus*, although its function in saliva is currently unknown [63].

Additional protease inhibitors upregulated in the *O*. *moubata* sialotranscriptome that could contribute to the antihaemostatic potential of tick saliva are alpha-2 macroglobulin, carboxipeptidase inhibitors, cystatin-2, several serine protease inhibitors (serpins) and a thyropin (Table 7).

Our results for alpha-2 macroglobulin parallel those of Saravanan et al. [91], who demonstrated that this protein is expressed in salivary glands of *O*. *moubata* and that their mRNA levels were upregulated upon blood meals. These authors suggest that, besides the functions of alpha-2 macroglobulin in immune defence, it could play a function as anticoagulant in saliva in synergy with the platelet aggregation inhibitors ornithodorin and moubatin [92].

A carboxypeptidase inhibitor from the tick *Rhipicephalus bursa* has been proved to stimulate fibrinolysis, contributing to the maintenance of host blood fluidity and facilitating blood ingestion [93]. In *O*. *moubata*, five highly upregulated (log_2_FC = 10.11) transcripts annotated as carboxy peptidase inhibitors have been found, which could perform functions similar to that observed for ixodid ticks.

Cystatins are inhibitors of cysteine proteases. They have been identified in saliva and midgut of *O*. *moubata*, where they play functions in blood digestion, haem detoxification and modulation of the host immune response [12,94,95]. We found four upregulated transcripts annotated as cystatin-2 that could act as immune modulators and suppress host adaptive immune response [95].

Thyropin is a cysteine protease inhibitor present as a repeat domain in human thyroglobulin. It has been identified in midgut and salivary glands of ixodids and several *Ornithodoros* tick species, but at present, its function is unknown [50,63,83,96].

#### Other protein families represented in the upregulated transcriptome

Acid and basic tail proteins are molecules with unknown function and abundantly found in salivary transcriptomes of ixodid and argasid ticks, suggesting that they play important roles in blood feeding [60,63,65,66]. In *O*. *moubata*, 44 and 48 transcripts have been annotated as different members of acid and basic tail protein families, respectively, reaching a joint expression level that is 3% of the expression of the upregulated transcriptome at 7 days after feeding (SG7) (Table 8, Fig 6A).

**Table 8:** Acid and basic tail proteins, vitellogenins, proteins with phospholipase A2 activity, a member of the 7DB family and proteins involved in immunity and defense. Transcripts annotated and differentially upregulated in the *O*. *moubata* sialotranscriptome 7 days after feeding (SG7 vs SG0). The number of transcripts, the average expression level (RPKM) in each physiological condition (SG0, SG7) and the logFC value range reached by the upregulated transcripts are shown.

Description	Accession	N° transcripts	RPKM SG0	RPKM SG7			logFC
**Acid tail proteins**							
Acid tail salivary protein	ABR23355, ABR23361 ACB70369, ACB70371	24	1,019.42	15,778.23			1.49–6.65
ATSP	ABI52732	20	305.79	4,266.36			2.14–4.11
**Basic tail proteins**							
BTSP	ABI52632, ACB70321 ACB70322, ACB70323 ACB70365, DAA34075	35	1,202.23	19,329.81			1.29–4.49
Salivary secreted basic tail protein	ABR23390, ACB70313	13	137.38	909.85			2.05–2.89
**Vitellogenins**							
Vitellogenin	AAW78557, BAH02666 ABS88989, B7QJ67	61	0.07	285.18			10.48–12.59
Vitellogenin-1	AGQ56698, AGQ57039 ASB34115	12	0.37	710.76			7.78–14.48
Vitellogenin-2	AXP34688	1	5.44	42.90			2.98
**Phospholipase A2 activity**							
Phospholipase A2	ABR23453, AGJ90343 ISCW014959,ISCW020728 ISCW020729, ACB70350 ACB70398, ACB70400	90	597.35			3,259.97	1.01–4.60
Putative salivary secreted peptide	AAT92118.1	1	1.70			5.03	1.56
**7DB Family**							
7DB family member	AGJ90344	30	639.71			8,623.81	1.53–4.66
**Immunity and defense**							
Complement C3	XP_021003824, XP_023225952 KFM60589, KFM76917 KFM82087, BAK64109	12	16.60		86.13		1.62–3.04
Complement component C2/Bf	ATP66484	4	45.16		149.92		1.59–1.79
Complement inhibitor	AAT65682, ACI46626 ACI46628	37	1,270.18		26,312.54		1.44–5.23
Defensin	ABI52686	3	2.08		12.31		2.05–2.67
Lysozyme	AAL17868, XP_011199991	23	136.47		5,273.35		2.05–5.82
Spatzle alternatively spliced	B7QFC1	1	3.03		6.71		1.15
Toll, putative	B7PUU4	7	3.80		13.04		1.17–3.17

Vitellogenin is a precursor of vitellin, which is essential for egg development and oviposition; in the midgut, it is also involved in lipid transport and haem detoxification [97,98]. It was thought that vitellogenin was synthesised in tick midgut and fat body only, but recently it has been found differentially overexpressed after feeding in the sialome of several tick species, suggesting that it is also synthesised in the salivary glands [59, 99]. The function of this protein in saliva and tick feeding is not demonstrated hitherto. Since the haem group has proinflammatory activity, it may be that the vitellogenin secreted to the saliva contributes to a reduction of the free haem concentration and its proinflammatory effects in the feeding lesion [99,100]. Additionally, since part of the tick saliva is ingested with the blood, it could be speculated that the ingested vitellogenin would also contribute to reduce the haem excess produced by enterocytes during the first phases of the blood digestion [12]. In *O*. *moubata*, we annotated 74 vitellogenin-upregulated transcripts at 7 days after feeding with log_2_FC values remarkably higher than in the remaining groups (up to 14.48; Table 8). It is also worth mentioning that 14 days after feeding there were 83 upregulated vitellogenin transcripts, representing up to 5% expression of the upregulated transcriptome at 14 days post-feeding (Fig 6B). These results, and the finding of vitellogenin protein in the saliva proteome [23], indicate that vitellogenin is abundantly synthesised in the salivary glands and secreted into the feeding lesion, suggesting a relevant role of this protein in tick feeding.

Phospholipases A2 (PLA2s) are a protein superfamily that includes the secreted PLA2 family, the latter being important components of some animal venoms and tick saliva [65,101]. Secreted PLA2s participate in numerous physiological processes including regulation of host inflammatory and defensive responses as well as novel signalling and cellular communication pathways [102]. More recently, a PLA2 from *O*. *moubata* was shown to act as an antagonist ligand for host P-selecting, inhibiting the haemostatic and pro-inflammatory processes started after the expression of P-selecting in the damaged vascular endothelium of the host [22]. A total of 91 transcripts of PLA2 or of proteins with this enzymatic activity were annotated in the *O*. *moubata* sialotranscriptome, which jointly represented up to 0.5% of the expression of the upregulated transcriptome at 7 days after feeding (SG7), highlighting the importance of the antiheamostatic and antiinflammatory functions played by this protein in tick feeding (Table 8, Fig 6A).

The 7DB protein family is unique to argasid ticks, and its functions are currently not known [20,67]. We annotated 30 upregulated transcripts as a 7DB family member, which has already been identified by Manzano-Román et al. [22] in a NAPPA protein array constructed from an *O*. *moubata* salivary gland cDNA expression library (Table 8).

Salivary transcriptomes of haematophagous insects and ticks show the presence of transcripts involved in defence and immune mechanisms [65]. In this *O*. *moubata* sialotranscriptome, 87 upregulated transcripts were annotated as immunity-related, representing 4.87% of the sialotranscriptome expression at 7 days after feeding (Table 8, Fig 6A). These transcript sequences are annotated as complement component C3 (12 transcripts), complement component C2/Bf (4 transcripts), complement inhibitor (37 transcripts), defensin (3 transcripts), lysozyme (23 transcripts) and two additional molecules involved in the innate immunity response, Spaetzle and toll proteins [103,104]. These complement components C3 and C2/Bf might play a role in protecting *O*. *moubata* ticks against yeast and *Borrelia* sp. infections, as has been already demonstrated in *Ixodes ricinus* [105]. Lysozyme and defensin are important antimicrobial molecules abundantly found in invertebrates, including *Ornithodoros* ticks, where they were upregulated in the midgut in response to blood feeding and digestion [12,14,63]. Lysozyme is effective against gram-positive bacteria and defensin against both gram-positive and-negative bacteria [99].

### Relevance for public health of the current research

Tick-borne human relapsing fever (HRF) is a severe neglected tropical disease widely distributed throughout many countries of East, Central and Southern mainland Africa and Madagascar. In this area HRF is produced by *Borrelia duttoni* and transmitted by *Ornithodoros moubata* ticks. In some of these countries, such as Tanzania and Ruanda, HRF is hyperendemic. There, HRF shows high annual incidences in children under one year and reaches perinatal mortality rates as high as 43.6% [6,106]. In these countries, *O*. *moubata* is found in nature, associated to warthogs and other wild hosts inhabiting burrows, but also in anthropic environments, colonizing the inside of human houses and domestic animal premises, which greatly contributes to the transmission and persistence of HRF in the affected areas. Thus, to be effective, any program aimed at the prevention and control of HRF shall require the elimination of at least the anthropic populations of this argasid, and tick vaccines seem to be the most promising strategy as an alternative to the application of chemical acaricides [9].

In the current research we have approached the identification of potentially protective candidate antigens for vaccine development by selecting and analysing several functional groups and families of tick salivary proteins that have important functions (either predicted or experimentally demonstrated) in biological processes related to blood feeding. These proteins include several clades of lipocalins and a range of metalloproteases, protease inhibitors, phospholipases A2, apyrases and vitellogenins, which significantly increase the current repertory of protective candidate antigens from argasid ticks. In fact, some of the antigens that have already demonstrated partial protective value against *O*. *moubata* in animal immunization trials belong to one of these families [9].

Interestingly, the selected protein groups and families show a high degree of functional redundancy as most of their members act as anticoagulants or inhibitors of platelet aggregation or as anti-inflammatory agents and inhibitors of the innate immune response; these functions contribute to maintain blood fluidity, to avoid the deleterious effects of inflammation and also allows feeding ticks to pass unnoticed by the host. This functional redundancy clearly indicates which host defensive responses must be necessarily abrogated by the tick to be able to fed, namely blood clotting, platelet aggregation and inflammation. Moreover, functional redundancy underscores the notion that targeting individual tick antigens will probably not be enough to reach total protection (for example, complete blocking of tick feeding) and highlights the need of simultaneously targeting several functionally redundant tick antigens to completely abolish the involved tick anti-defensive mechanism and reach full protection; in other words, functional redundancy points to the necessity of developing multiantigenic vaccines rather than single antigen vaccines for the control tick infestations and tick-borne disease transmission.

In summary, this research provides a range of promising candidate antigens that may be included in development of vaccines for the control of *O*. *moubata* infestations, which will positively impact in the prevention of tick-borne diseases of public and veterinary health significance as human relapsing fever and African swine fever.

## Conclusions

We assembled *de novo* the transcriptome of *O*. *moubata* salivary glands (sialotranscriptome) using next-generation sequencing technologies, resulting in 71,194 transcript clusters and 41,011 annotated transcripts, which represents 57.6% of annotation success. The annotated transcripts corresponded to thousands of protein-coding sequences and unveiled large multigene protein families, many of them conserved between argasid and ixodid ticks.

The complexity and functional redundancy observed in the sialotranscriptome of *O*. *moubata* are comparable to those of the sialomes of other argasid and ixodid ticks, with lipocalins, metalloproteases and protease inhibitors as the protein families/groups most abundantly represented.

These data significantly enlarge the limited repertory of argasid salivary protein-coding sequences currently available and contribute to the rapidly increasing number of tick salivary protein-coding sequences deposited in public databases.

Differential gene expression analysis along the trophogonic cycle showed that most of the salivary upregulated gene expression takes place in the first 7 days after feeding, with low significance between 7 and 14 days. This allows that, during the off-host period, the tick can complete the replacement of all the salivary bioactive proteins consumed during feeding to be ready for the following blood meal. Since *O*. *moubata* and the argasid ticks typically are fast feeders, they do not need to change their saliva composition along the feeding process, in contrast to ixodid ticks, which experience the so-called “sialome and saliva switching”, adding higher complexity to their sialomes.

Functional GO term and metabolic pathway enrichment analysis of the differentially upregulated genes after feeding resulted in several groups of genes that were abundantly expressed and that code for proteins functionally related to blood ingestion and modulation of the host defensive responses, including lipocalins, metallopeptidases, protease inhibitors, phospholipases, apyrases, vitellogenins, proteins associated with immunity and defence and tick-specific families such as 7DB, as well as acid and basic tail proteins.

Because of their overexpression and functions as antihaemostatic and/or anti-inflammatory factors, several of the identified lipocalins, metalloproteases, protease inhibitors, phospholipases A2, apyrases and vitellogenins can be interesting candidate protective antigens for the development of vaccines against *O*. *moubata*, which significantly increase the current repertory of protective candidate antigens from argasid ticks.

As a whole, these proteins exhibit great functional redundancy since most of them act as anti-clotting, anti-platelet aggregation and anti-histaminic factors. This redundancy clearly indicates which host defensive responses must be necessarily abrogated by *O*. *moubata* to be able to fed, and underlines the necessity of developing multiantigenic vaccines targeting several functional candidate orthologues in order to completely abolish the involved tick anti-defensive mechanism and reach full protection.

This transcriptome provides a valuable reference database for ongoing proteomics studies of the salivary glands and saliva of *O*. *moubata* to confirm and expand previous data on the *O*. *moubata* sialoproteome, which will in turn validate this transcriptome assembly.

Integration of transcriptomic and proteomic data will allow the selection of antigenic candidates that may be useful for developing vaccines for the control of *O*. *moubata* infestations, which will in turn contribute to the prevention of tick-borne diseases of public and veterinary health significance such as human relapsing fever and African swine fever.

## Supporting information

S1 Table Metrics of the transcriptome assembly of each sample. (XLSX)

S2 Table Sialotranscriptome annotated of *Ornithodoro moubata*. SG0_1 and SG0_2, salivary glands from unfed ticks; SG7_1 and SG7_2 salivary glands from ticks at 7 days after feeding; SG14_1 and SG14_2 salivary glands from ticks at 14 days after feeding.
(XLSX)

S3 Table Pfam domain occurrences in the *Ornithodoro moubata* predicted proteins. (XLSX)

S4 Table Biological pathways identified in the salivary glands of *Ornithodoro moubata*. (XLSX)

S5 Table Differential gene expression in comparisons SG7 *vs* SG0, SG14 *vs* SG7 and SG14 *vs* SG0. SG0, salivary glands from unfed ticks; SG7 and SG14, salivary glands from ticks at 7 and 14 days after feeding, respectively
(XLSX)

S6 Table Significantly enriched GO terms assigned to the differentially expressed transcripts in comparisons SG7 *vs* SG0, SG14 *vs* SG0 and SG14 *vs* SG7. SG0, salivary glands from unfed ticks; SG7 and SG14, salivary glands from ticks at 7 and 14 days after feeding, respectively. NumInCat, total number of sequences in the category. NumDEInCat, number of enriched sequences in each category.
(XLSX)

S7 Table Significantly enriched metabolic pathways of differentially expressed transcripts in comparisons SG7 *vs* SG0, SG14 *vs* SG0 and SG14 *vs* SG7. SG0, salivary glands from unfed ticks; SG7 and SG14, salivary glands from ticks at 7 and 14 days after feeding, respectively. NumInCat, total number of sequences in the pathway. NumDEInCat, number of enriched sequences in each pathway.
(XLSX)

S1 Dataset Numerical values used to generate the Figures. (XLSX)
